# Recent advances in proteomic workflows to interrogate the SUMOylome in plants

**DOI:** 10.1111/nph.70176

**Published:** 2025-05-06

**Authors:** Kishor D. Ingole, Elizaveta Alekseeva, Kathryn S. Lilley, Ari Sadanandom

**Affiliations:** ^1^ Department of Biochemistry University of Cambridge Cambridge CB2 1RX UK; ^2^ Department of Biosciences Durham University Durham DH1 3LE UK

**Keywords:** posttranslational modifications (PTMs), proteomic workflows, Small Ubiquitin‐like MOdifier (SUMO), stress response, SUMOylation

## Abstract

Protein posttranslational modifications (PTMs) are vital for regulating protein functions. SUMOylation, a PTM essential for plant survival, involves attaching a Small Ubiquitin‐like MOdifier (SUMO) to lysine residues of target proteins. SUMOylation influences stress tolerance, cell proliferation, protein stability, and gene expression. While well studied in mammals and yeast, SUMOylation studies in plants are scarce, as the identification of SUMOylated proteins and the specific modification sites is challenging. Deciphering the plant SUMOylome is essential for understanding stress response mechanisms. Advanced proteomic techniques are necessary to map these complex protein modifications. This article offers insights into the workflows employed for probing the SUMOylome. We analyze how current technological approaches have advanced our understanding of SUMOylation and highlight limitations that currently impede comprehensive mapping of SUMO signaling pathways.

**Table:** 

	Contents	
	Summary	90
I.	Introduction	90
II.	Qualitative SUMOylome studies using 6xHis‐SUMO1^H89R^ and 3D gel proteomics	91
III.	Quantitative SUMOylome studies using 6xHis‐SUMO1^H89R^	93
IV.	Quantitative SUMOylome using lysine null *K0‐SUMO1*	93
V.	Conclusion and perspectives	94
	Acknowledgements	95
	References	95

## I. Introduction

More than 500 posttranslational modifications (PTMs) of proteins are known to exist, playing an important role in protein stability, catalysis, activity, protein localization, and in changing the interactome (Vu *et al*., 2018; Keenan *et al*., 2021). SUMOylation is a versatile PTM in which a Small Ubiquitin‐like MOdifier (SUMO), a *c*. 11‐kDa protein, is covalently attached to lysine (K) of the target proteins, commonly occurring at the conserved ᴪKXD/E motif, and it may also occur at nonconsensus motifs (Chang *et al*., 2018). A given protein can be conjugated by one or more SUMO molecules, defining the mono‐, multi‐, or polySUMOylation status of that protein. Genetic screens have proven that SUMOylation is essential for plant survival; it affects many aspects of plant metabolism, such as biotic and abiotic stress tolerance, cell proliferation, protein stability, and gene expression (Park *et al*., 2011; Han *et al*., 2021; Ingole *et al*., 2021b; Srivastava *et al*., 2021; Ghosh *et al*., 2024). While investigating SUMOylation in plants has proven challenging, understanding of the SUMOylome (the collection of SUMO conjugates) in plants is an important objective, considering the importance of the SUMO modification in stress response pathways.

The SUMOylation pathway operates through a three‐step, enzyme‐catalyzed cascade. In the model plant *Arabidopsis thaliana*, this process includes the SUMO‐activation enzyme E1, which is a heterodimer of SAE1a/b and SAE2, a single E2 SUMO‐conjugation enzyme (SCE1), and E3 ligation mediated by the SAP‐MIZ domain‐containing SIZ1 and HIGH PLOIDY2 (HPY2) also called METHYL METHANESULFONATE‐SENSITIVE21 (MMS21) (Park *et al*., 2011). Multiple (*c*. 16) deSUMOylating proteases are present, making SUMOylation a reversible PTM. The Arabidopsis genome encodes eight SUMO isoform proteins, with SUMO1 and SUMO2 sharing high sequence identity and being the most abundantly expressed variants.

SUMOylation plays a significant dual role in its interaction with ubiquitination, by either protecting proteins from ubiquitination or exposing lysines for ubiquitination and thus directing proteins for proteasomal degradation (Geoffroy & Hay, 2009).

**Fig. 1:**
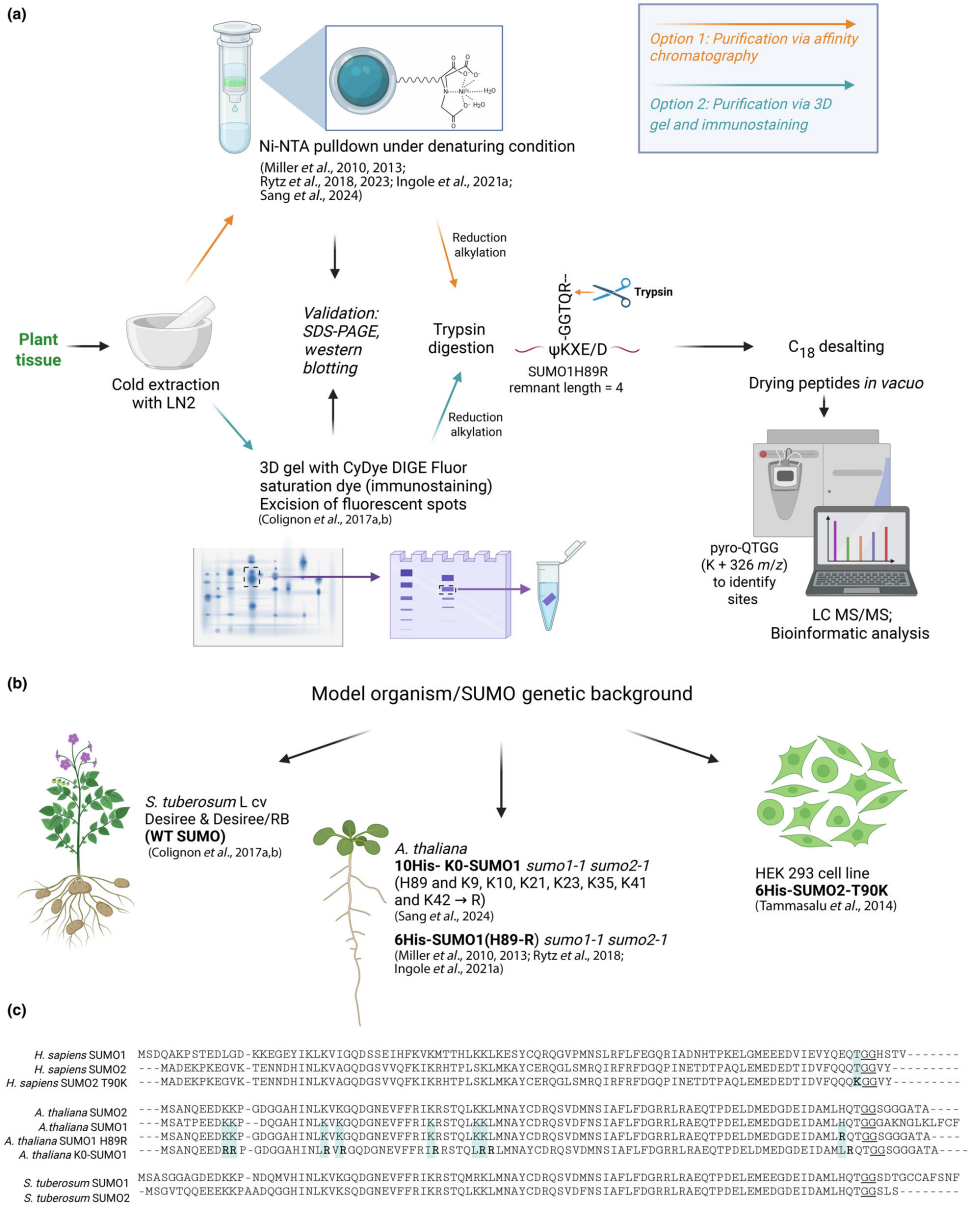
Schematic representation of the workflow for sample preparation and enrichment of SUMO1‐conjugates for liquid chromatography mass spectrometry (LC‐MS)/MS analysis. (a) Overview of the proteomic workflow, including nickel–nitrilotriacetic acid (Ni‐NTA)‐based enrichment of 6xHis‐SUMO1^H89R^, 2D/3D gel‐based immunostaining, and subsequent LC‐MS/MS identification. (b) Illustration of various model organisms utilized to study the SUMOylome, showcasing their diverse genetic backgrounds. (c) Depiction of SUMO1 and SUMO2 amino acid sequences (both wild‐type and modified versions) used to develop transgenic lines for SUMOylome investigation. This figure was created in BioRender (https://BioRender.com/ys5zpu3).

Mass spectrometry (MS) is widely used for deep proteome analysis; however, MS‐based analysis of PTMs comes with significant challenges. Mass spectrometry analysis of ubiquitin conjugates using shotgun proteomics involves the digestion of ubiquitinated proteins to peptides using Trypsin. The tryptic digestion of ubiquitinated proteins generates peptides from the target proteins that have a diGly (GG) remnant from the C‐terminus of ubiquitin on the lysine (K) at the site of attachment, which are relatively easily analyzed by MS. However, upon tryptic digestion of SUMO conjugates, a *c*. 25 amino acid remnant of SUMO remains covalently attached to lysine side chains of the target protein, leading to complex mass spectra that are difficult to interpret. To overcome this problem, the introduction of mutations near the C‐terminus of the SUMO protein which create a site amenable to proteolytic digestion leaving shorter remnant sequences, or multi‐step purification and enrichment steps, is necessary. A brief overview of potential experimental approaches employed in plants is illustrated in Fig. 1. This Tansley insight provides an overview of various experimental strategies and proteomic workflows employed to investigate the SUMOylome across different plant species, highlighting their applications and limitations.

## II. Qualitative SUMOylome studies using 6xHis‐SUMO1^H89R^ and 3D gel proteomics

Recent evidence suggests that SUMO conjugates become enriched under specific stresses, such as heat, oxidative stress, or infection (Miller *et al*., 2010; Ingole *et al*., 2021a). Comparative quantitative proteomics coupled with liquid chromatography (LC‐MS/MS) allows profiling of changes in the SUMOylome during stress (Miller *et al*., 2013; Hendriks *et al*., 2018; Sang *et al*., 2024). Enriching SUMO conjugates typically involves genetically modifying plants to express a tagged version of SUMO1, replacing the endogenous gene. Miller *et al*. (2010) implemented this approach by engineering an *A. thaliana* model with a 6xHis‐SUMO1^H89R^ protein that substituted the native SUMO1 (Fig. 1a–c). Upon the digestion of SUMOylated proteins with trypsin, the four C‐terminal amino acids of the modified SUMO1 leave a pyro‐QTGG remnant (K+ 326 *m/z*) at the site of conjugation of the target protein, which can be identified by subsequent mass spectrometric analysis. The authors investigated SUMOylation under stress by subjecting plants to heat (37°C) or oxidative stress (50 mM H_2_O_2_). Following the enrichment of SUMO1 conjugates using nickel–nitrilotriacetic acid (Ni‐NTA) affinity and anti‐SUMO1 immunoaffinity techniques, LC‐MS analysis identified 357 putative SUMO1 targets. Seventy‐six percent of these SUMOylated proteins were associated with nuclear functions, highlighting their significant role in gene expression regulation.

SUMOylation also plays a role in plant infection responses (Orosa *et al*., 2018; Verma *et al*., 2018). Ingole *et al*. studied Arabidopsis SUMOylation under *Pseudomonas syringae* pv tomato (PstDC3000) infection using the 6xHis‐SUMO1^H89R^ in wild‐type (WT) (Columbia) and *srfr1‐4* autoimmune mutant. The *srfr1‐4* line, deficient in SUPPRESSOR OF rps4‐RLD1 (SRFR1; a regulator preventing immune mis‐priming), exhibited increased SUMO1 conjugates. The SUMO1 conjugates were analyzed in four experimental lines (His‐SUMO1^H89R^ *sum1‐1 sum2‐1* untreated, His‐SUMO1^H89R^ *sum1‐1 sum2‐1* mock treated, His‐SUMO1^H89R^ *sum1‐2 sum2‐1 srfr1‐4* untreated, and His‐SUMO1^H89R^ *sum1‐1 sum2‐1 PstDC3000* infected). Enriched conjugates were subjected to LC‐MS/MS leading to the identification of 261 SUMO conjugates. Gene Ontology (GO) analysis again highlighted nuclear‐localized proteins as targets of SUMOylation involved in a broad range of functions, including transcription, RNA processing, detoxification, and chromatin remodeling. STRING database analysis revealed extensive protein interaction networks, further supporting SUMOylation's critical role in rapid stress‐induced metabolic reprogramming (Ingole *et al*., 2021a).

Posttranslational modification research aims to create a comprehensive subcellular map of enzymes that bring about the modification and their targets. Rytz *et al*. (2018) probed mutants for two known SUMO ligases, SIZ1 and MMS21, known for their role in stress tolerance and maintenance of DNA integrity, respectively, for differences in the SUMOylome. Each mutant was analyzed in normal and heat stress conditions. All plants used in the study were expressing the 6xHis‐SUMO1^H89R^ to allow the same purification method as described in Miller *et al*. (2010) and subjected to MS analysis. A considerable loss of diversity in SUMO conjugates was detected in the *siz1‐2* background compared with that of WT plants, both in heat‐stressed and in unstressed conditions. Altogether, more than 1000 putative SUMO1 targets were identified. GO analysis confirmed that SIZ1 conjugates are enriched in stress regulators. The authors identified *c*. 40 SUMO modification sites, including three lysine residues (K100, K479, and K488) on the SUMO E3 enzyme SIZ1. To explore their functional significance, the authors generated a SIZ1 3K‐R mutant (K‐to‐R substitutions) and analyzed its effects in plants. Unfortunately, no MMS21 targets could be identified with confidence using this approach, suggesting that it modifies only a few low‐abundance proteins, highlighting the need to improve the workflow further to allow detection of low‐abundance species. Genetic mutants of SUMO ligases can aid in identifying enzyme–substrate relationships. However, SUMO conjugation is influenced by more than conjugase activity. In Arabidopsis, 16 SUMO proteases, divided into two classes, regulate SUMOylation. Class I ubiquitin‐like proteases (ULPs) (e.g. OTS1/2, ESD4 and ELS1) are involved in SUMO maturation and deconjugation, while Class II proteases (DeSumoylating Isopeptidase (DeSI) family) focus solely on deSUMOylation (Srivastava *et al*., 2021). Unlike ubiquitination, which depends more on E3 ligases, SUMOylation is regulated by both ligases and proteases, adding an unexplored layer of complexity in plant biology.

An alternative approach to identifying SUMO‐conjugated proteins was introduced by Colignon *et al*. (2017a,b) who used a 3D gel separation and blotting technique to isolate and characterize 39 nonredundant SUMO conjugates in *Solanum tuberosum* (potato) plants and identify potential significance of SUMOylation during infection. The workflow involved resolving leaf extracts on a 2D gel and visualization of SUMO conjugates with a CyDye DIGE fluor saturation dye‐conjugated anti‐SUMO1 antibody, excision of fluorescent spots, and repeated resolution and visualization on a 1D gel, yielding a ‘3D’ resolution. While Arabidopsis is considered a better model system for plant research due to the ease of genetic modification, research performed by Colignon *et al*. is valuable as it shows the importance of SUMOylation directly in a non‐genetically modified food crop. An issue with this approach is that 2D gels are an imperfect method of separating proteins as many proteins will have similar isoelectric points and molecular weights, leading to co‐migration on 2D gels where these properties are exploited.

## III. Quantitative SUMOylome studies using 6xHis‐SUMO1^H89R^

Miller *et al*. (2013) determined the changes in the extent of SUMOylation using a quantitative proteomic workflow to analyze 172 SUMO targets during the heat‐induced stress in Arabidopsis, refining the protocol to minimize sample‐to‐sample variation by using 4‐plex iTRAQ isobaric tags. Isobaric tags, such as iTRAQ (Ross *et al*., 2004) and tandem mass tags (TMT; Thompson *et al*., 2003), share the same mass but fragment during MS–MS to liberate reporter ions of distinct sizes specific for each tag that can be identified postanalysis. In this way, samples can be multiplexed, now up to 35‐plex samples (Zuniga *et al*., 2024) in the same experiment, reducing variation caused by instrumental differences between runs. However, accurate quantification is challenged by variations in isobaric tag labeling efficiency, background interference during precursor ion selection in complex samples, and inconsistent sample preparation, particularly with the three‐column purification steps (sequential Ni‐NTA, anti‐SUMO1/2 affinity, and Ni‐NTA chromatography) required for SUMO1 conjugates before MS analysis. To mitigate these issues, in the study of Miller *et al*. (2013), an internal control was introduced by adding equal amounts of purified recombinant 6xHis‐SUMO2 protein to the samples. Since the used Arabidopsis mutant (6His‐SUMO1^H89R^ *sum1–1 sum2–1* line) lacks endogenous SUMO2, both SUMO1 and SUMO2 were efficiently recognized by the anti‐SUMO1 antibody, facilitating co‐purification. During MS fragmentation, SUMO2 produces unique peptides distinguishable from SUMO1, making it an effective internal control. Analysis of 172 SUMO substrates during and after heat shock (37°C) indicated that stress primarily enhanced the abundance of preexisting conjugates rather than introducing modifications to new targets.

## IV. Quantitative SUMOylome using lysine null *K0‐SUMO1*

Using the SUMO1^H89R^ approach, only a limited number of SUMO modification sites (*c*. 80) have been identified in *Arabidopsis*. In animal systems, a more comprehensive, site‐specific proteome analysis has employed the use of a N‐terminally His‐tagged lysine‐null SUMO2 variant (10xHis‐SUMO2‐K0‐Q87R). This method has been successfully used to identify thousands of SUMOylation sites in mammalian cells (Hendriks & Vertegaal, 2016; Hendriks *et al*., 2017, 2018). It formed the basis for the highly sensitive SUMOylome detection protocol recently developed by Sang *et al*. (2024) in which the *K0‐SUMO1* Arabidopsis transgenic lines are generated in a *sum1 sum2* mutant background in which all seven Lys residues (K9, K10, K21, K23, K35, K41, and K42) and His89 are substituted with Arg in SUMO1 (Fig. 1c). The K0‐SUMO1 construct also harbors a 10xHis tag at the N‐terminus to facilitate its enrichment under denaturing conditions using Ni‐NTA beads. Upon digestion with Lys‐C, target proteins are converted to peptides, but SUMOylated peptides still retain modification with intact SUMO1 by virtue of it lacking Lys‐C (a protease that cleaves at the C‐terminal side of lys amino acids) digestion sites. This enables a second round of enrichment of SUMOylated peptides as the N‐terminal tag of the K0‐SUMO1 remains intact. The workflow thus includes two Ni‐NTA enrichment steps with Lys‐C digestion in between, followed by trypsin digestion and LC‐MS/MS analysis. The enrichment workflow was enhanced by using plant‐specific buffers, SUMO protease inhibitors to prevent deSUMOylation, and a two‐step elution with imidazole and ethylenebis(oxyethylenenitrilo)tetraacetic acid (EGTA), improving K0‐SUMO1 conjugate recovery. However, it is important to note that the K0 variant leads to the inability to detect branched and polySUMO targets as all the internal lysines are mutated. As a result, 2200 unique SUMOylation sites that mapped to *c*. 1300 putative SUMO1 acceptors were identified, a significant improvement compared with previous studies involving affinity purification. The authors further conducted *in vitro* validation of nine SUMO targets (CPK3, CIPK23, EHD, CPK18, TIL1, EIN3, HSFA3, EROI, and PEP1) using a reconstituted *Escherichia coli* strain expressing the Arabidopsis SUMO machinery (SUMO, E1, and E2; Okada *et al*., 2009), confirming their SUMOylation. Additionally, they examined the effects of lysine‐to‐arginine mutations (CPK3^K318R^, HSFA3^K242R^, and PEP1^K3R^), demonstrating that these mutations resulted in reduced SUMOylation *in vitro*. The change in the SUMOylome in response to heat stress was also assayed with quantitative MS using TMT isobaric tags. In total, 435 SUMOylated substrates were identified, with 128 showing an increase in SUMOylation upon heat stress and 16 showing a decrease. Targets that exhibited an increase in SUMOylation included heat‐shock transcription factors, components of the SUMOylation pathway, transcription factors, and nuclear‐localized proteins, while ribosome components and ribonucleoprotein complexes showed a net decrease in SUMOylation.

SUMO can undergo secondary modifications, such as SUMOylation and ubiquitylation, although the roles of these additional modifications remain unclear. To study their function, transgenic Arabidopsis was generated replacing essential SUMO1 and SUMO2 isoforms with a K0‐SUMO1 that prevents further modifications. These plants developed normally and had nearly unchanged SUMOylation profiles during heat shock, but exhibited altered sensitivities to salt, oxidative stress, DNA‐damaging agents, and hormone signaling, thus highlighting potential roles for secondary SUMO modifications in stress response, DNA repair, and hormone regulation (Rytz *et al*., 2023). These studies allowed the development of important controls for the work by Sang *et al*., (2024) where K0‐SUMO1 mutants were used to isolate SUMOylated protiens form Arabidopsis.

Purification and enrichment of SUMO conjugates is a critical step in the workflow, and improving its specificity and recovery rate is largely responsible for the quality of the MS data and the identification of potential targets and modification sites. Anti‐K‐ε‐GG peptide, antibody affinity chromatography combined with Ni‐NTA chromatography has been recently applied to purify SUMO‐modified peptides from animal cells expressing 6xHis‐SUMO^T90K^, which upon digestion with trypsin, leave a diGly (−GG) remnant at the attachment site lysine of the target protein (Tammsalu *et al*., 2014). Anti‐K‐ε‐GG antibodies were first used to probe the ubiquitinome, as the diGly remnant is naturally present in trypsin‐digested ubiquitin conjugates (Udeshi *et al*., 2013). SUMO conjugate digests do not have a diGly remnant naturally; however, SUMO can be mutated to generate a diGly remnant on modified peptides when digested with Lys‐C and Glu‐C (cleaves at the C‐terminus of either aspartic (D) or glutamic acid (E) residues) enzymes instead of trypsin. Tammasalu *et al*. first developed this method in HEK 293 cells by using a 6xHis‐SUMO^T90K^ substitution near the C‐terminus (Fig. 1c). The first enrichment step consists of a Ni‐NTA affinity purification, followed by Lys‐C digestion on a 30 kDa filter. Larger proteins left on the filter are further digested by Glu‐C and eluted separately. Glu‐C and Lys‐C digested peptides are then affinity‐purified using the anti‐K‐ε‐GG antibody. This protocol reduced the complexity of final peptide samples by *c*. 500‐fold, making MS analysis easier and more efficient (Tammsalu *et al*., 2014, 2015). This protocol has not been employed to enrich SUMO‐modified peptides from any plant species to date. The K‐ε‐GG peptide antibody enrichment protocol for plant samples may need extensive optimization due to high concentrations of waxes, polyphenols, and other metabolites in the samples that might interfere with the K‐ε‐GG antibody binding to SUMO‐modified peptides.

## V. Conclusion and perspectives

SUMOylation is a versatile PTM that plays a critical role in regulating protein stability, activity, and localization, as well as plant responses to various stresses. Despite its significance, studying SUMOylation in plants remains challenging due to the complexity of the SUMOylome and the limitations of current analytical techniques. Advancements in methodologies, such as the development of tagged SUMO constructs and refined MS protocols, have enabled deeper insights into SUMOylation dynamics. Quantitative studies utilizing iTRAQ and TMT labeling have revealed stress‐specific SUMOylation patterns, highlighting its regulatory role during heat and oxidative stress. Techniques such as 2D/3D gel‐based proteomics present an approach for identifying SUMO targets in nonmodel crop plants like potato, which are challenging to genetically modify. The summary of proteomic studies on plant SUMOylation, including the number of identified putative SUMO targets and modification sites, is listed in Table 1.

**Table 1:** Summary of proteomic studies on plant SUMOylation, including the number of identified putative SUMO targets and modification sites.

Study	Targets identified	SUMOylation sites identified	Model system with their genetic background
Miller *et al*. (2010)	357	17	*Arabidopsis thaliana* (6xHis‐SUMO1^H89R^ in *sum1‐1 sum2‐1*)
Miller *et al*. (2013)	172	–	*A. thaliana* (6xHis‐SUMO1^H89R^ in *sum1‐1sum2‐1*)
Colignon *et al*. (2017a)	39	–	*Solanum tuberosum* L. cv Desiree (wild‐type)
Colignon *et al*. (2017b)	39	–	*S. tuberosum* L. cv Desiree (susceptible), *S. tuberosum* L. cv Desiree/RB (resistant)
Rytz *et al*. (2018)	1058	*c*. 40	*A. thaliana* (6xHis‐SUMO1^H89R^ in *sum1‐1 sum2‐1*; 6xHis‐SUMO1^H89R^ in *sum1‐1 sum2‐1 siz1‐2*; 6xHis‐SUMO1^H89R^ in *sum1‐1 sum2‐1 mms21‐1*)
Ingole *et al*. (2021a,b)	261	8	*A. thaliana* (6xHis‐SUMO1^H89R^ in *sum1‐1 sum2‐1*; 6xHis‐SUMO1^H89R^ in *sum1‐1 sum2‐1 srfr1‐4*)
Rytz *et al*. (2023)	109	–	*A. thaliana* (6xHis‐(M1R)‐SUMO1^H89R^ in *sum1‐1 sum2‐1*; 6xHis‐(M1R)‐K0(H89R) in *sum1‐1 sum2‐1*)
Sang *et al*. (2024)	1300	2235	*A. thaliana* (10xHis‐K0‐SUMO1 in *sum1‐1 sum2‐1*)

The selection of a proteomic approach for identifying SUMO targets and their modification sites largely depends on the plant species used. For genetically modifiable plant species, we strongly recommend incorporating a 10× His‐tag at the N‐terminus of SUMO. This enables the enrichment of SUMO conjugates under strong denaturing conditions, which is crucial due to the fragile nature of SUMOylation. The high activity of SUMO proteases can rapidly cleave SUMO from target proteins, making stabilization essential. It is also strongly recommended to use a plant protease inhibitor cocktail and N‐ethylmaleimide (NEM), which stabilizes SUMO conjugates by covalently modifying the sulfhydryl group of the catalytic cysteine on SUMO‐specific proteases. For MS analysis, introducing a K/R mutation at the C‐terminus of SUMO is important to generate a smaller SUMO footprint on modified peptides. For example, tryptic digestion of SUMOH89R results in a −QTGG remnant (K + 326 *m/z*), whereas SUMOK/RGG leaves a −GG remnant (K + 114 *m/z*), facilitating more precise detection using MS.

Despite rapid progress in the field, many aspects of SUMOylation remain unexplored. One is the cell‐type specificity of SUMO expression and modification. Investigating cell‐type‐specific SUMO targets requires generating transgenic lines that express a specific SUMO isoform under cell‐type‐specific promoters, enabling SUMO expression in targeted cell types, which is challenging to explore due to low protein abundance. Optimizing novel enrichment techniques, such as K‐ε‐GG antibody purification, is essential to selectively isolate SUMO‐modified peptides from complex peptide mixtures with high efficiency, allowing a reduction in sample complexity, enhancing the detection of SUMO sites, and improving the likelihood of identifying low‐abundance SUMO‐modified proteins.

An additional challenge that needs to be explored is the role of posttranslational C‐terminal processing of SUMO to expose diGly for conjugation during maturation (Bea *et al*., 2020). The remnant C‐terminal part after processing is thought to have a feedback regulatory role in SUMO gene expression. The open reading frame used in genetic constructs represents the ‘mature’ form of SUMO, making its expression in these constructs even more distinct from the native protein.

Future research should focus on mapping enzyme–substrate relationships by exploring new techniques, such as ubiquitin‐specific proximity‐based labeling (Ub‐POD) to identify E3 substrates (Mukhopadhyay *et al*., 2024) and also exploring cell‐type specificity of SUMOylation. In future, disruptive technologies that do not rely on MS, for example, nanopore approaches (Lan *et al*., 2024), and aptamer and antibody‐based approaches (https://www.nautilus.bio/platform/; Wik *et al*., 2021), will increase ease and throughput of large sample numbers and enhance our understanding of protein modifications.

## Competing interests

None declared.

## Author contributions

KDI, AS and KSL conceptualized the perspective. KDI and EA drafted the manuscript, while AS and KSL revised it. KDI and EA contributed equally as co‐first authors to this work.

## Disclaimer

The New Phytologist Foundation remains neutral with regard to jurisdictional claims in maps and in any institutional affiliations.

## References

[nph70176-bib-0001] Bea A , Kröber‐Boncardo C , Sandhu M , Brinker C , Clos J . 2020. The *Leishmania donovani* SENP protease is required for SUMO processing but not for viability. Genes 11: 1198. 33066659 10.3390/genes11101198 PMC7602377

[nph70176-bib-0002] Chang C‐C , Tung C‐H , Chen C‐W , Tu C‐H , Chu Y‐W . 2018. SUMOgo: prediction of sumoylation sites on lysines by motif screening models and the effects of various post‐translational modifications. Scientific Reports 8: 15512. 30341374 10.1038/s41598-018-33951-5 PMC6195521

[nph70176-bib-0003] Colignon B , Delaive E , Dieu M , Demazy C , Muhovski Y , Wallon C , Raes M , Mauro S . 2017a. Proteomics analysis of the endogenous, constitutive, leaf SUMOylome. Journal of Proteomics 150: 268–280. 27671789 10.1016/j.jprot.2016.09.012

[nph70176-bib-0004] Colignon B , Dieu M , Demazy C , Delaive E , Muhovski Y , Raes M , Mauro S . 2017b. Proteomic study of SUMOylation during *Solanum tuberosum–Phytophthora infestans* interactions. Molecular Plant–Microbe Interactions 30: 855–865. 28726589 10.1094/MPMI-05-17-0104-R

[nph70176-bib-0005] Geoffroy M‐C , Hay RT . 2009. An additional role for SUMO in ubiquitin‐mediated proteolysis. Nature Reviews Molecular Cell Biology 10: 564–568. 19474794 10.1038/nrm2707

[nph70176-bib-0006] Ghosh S , Mellado Sanchez M , Sue‐Ob K , Roy D , Jones A , Blazquez MA , Sadanandom A . 2024. Charting the evolutionary path of the SUMO modification system in plants reveals molecular hardwiring of development to stress adaptation. Plant Cell 36: 3131–3144. 38923935 10.1093/plcell/koae192 PMC11371177

[nph70176-bib-0007] Han D , Chen C , Xia S , Liu J , Shu J , Nguyen V , Lai J , Cui Y , Yang C . 2021. Chromatin‐associated SUMOylation controls the transcriptional switch between plant development and heat stress responses. Plant Communications 2: 100091. 33511343 10.1016/j.xplc.2020.100091 PMC7816078

[nph70176-bib-0008] Hendriks IA , Lyon D , Su D , Skotte NH , Daniel JA , Jensen LJ , Nielsen ML . 2018. Site‐specific characterization of endogenous SUMOylation across species and organs. Nature Communications 9: 2456. 10.1038/s41467-018-04957-4 PMC6018634 29942033

[nph70176-bib-0009] Hendriks IA , Lyon D , Young C , Jensen LJ , Vertegaal ACO , Nielsen ML . 2017. Site‐specific mapping of the human SUMO proteome reveals co‐modification with phosphorylation. Nature Structural & Molecular Biology 24: 325–336. 10.1038/nsmb.3366 28112733

[nph70176-bib-0010] Hendriks IA , Vertegaal ACO . 2016. A high‐yield double‐purification proteomics strategy for the identification of SUMO sites. Nature Protocols 11: 1630–1649. 27560170 10.1038/nprot.2016.082

[nph70176-bib-0011] Ingole KD , Dahale SK , Bhattacharjee S . 2021a. Proteomic analysis of SUMO1‐SUMOylome changes during defense elicitation in Arabidopsis. Journal of Proteomics 232: 104054. 33238213 10.1016/j.jprot.2020.104054

[nph70176-bib-0012] Ingole KD , Kasera M , Van Den Burg HA , Bhattacharjee S . 2021b. Antagonism between SUMO1/2 and SUMO3 regulates SUMO conjugate levels and fine‐tunes immunity. Journal of Experimental Botany 72: 6640–6658. 34145454 10.1093/jxb/erab296

[nph70176-bib-0013] Keenan EK , Zachman DK , Hirschey MD . 2021. Discovering the landscape of protein modifications. Molecular Cell 81: 1868–1878. 33798408 10.1016/j.molcel.2021.03.015 PMC8106652

[nph70176-bib-0014] Lan W‐H , He H , Bayley H , Qing Y . 2024. Location of phosphorylation sites within long polypeptide chains by binder‐assisted nanopore detection. Journal of the American Chemical Society 146: 24265–24270. 38986019 10.1021/jacs.4c03912 PMC11378271

[nph70176-bib-0015] Miller MJ , Barrett‐Wilt GA , Hua Z , Vierstra RD . 2010. Proteomic analyses identify a diverse array of nuclear processes affected by small ubiquitin‐like modifier conjugation in *Arabidopsis* . Proceedings of the National Academy of Sciences, USA 107: 16512–16517. 10.1073/pnas.1004181107 PMC2944710 20813957

[nph70176-bib-0016] Miller MJ , Scalf M , Rytz TC , Hubler SL , Smith LM , Vierstra RD . 2013. Quantitative proteomics reveals factors regulating RNA biology as dynamic targets of stress‐induced SUMOylation in Arabidopsis. Molecular & Cellular Proteomics 12: 449–463. 23197790 10.1074/mcp.M112.025056 PMC3567865

[nph70176-bib-0017] Mukhopadhyay U , Levantovsky S , Carusone TM , Gharbi S , Stein F , Behrends C , Bhogaraju S . 2024. A ubiquitin‐specific, proximity‐based labeling approach for the identification of ubiquitin ligase substrates. Science Advances 10: eadp3000. 39121224 10.1126/sciadv.adp3000 PMC11313854

[nph70176-bib-0018] Okada S , Nagabuchi M , Takamura Y , Nakagawa T , Shinmyozu K , Nakayama J , Tanaka K . 2009. Reconstitution of *Arabidopsis thaliana* SUMO pathways in *E. coli*: functional evaluation of SUMO machinery proteins and mapping of SUMOylation sites by mass spectrometry. Plant and Cell Physiology 50: 1049–1061. 19376783 10.1093/pcp/pcp056

[nph70176-bib-0019] Orosa B , Yates G , Verma V , Srivastava AK , Srivastava M , Campanaro A , De Vega D , Fernandes A , Zhang C , Lee J *et al*. 2018. SUMO conjugation to the pattern recognition receptor FLS2 triggers intracellular signalling in plant innate immunity. Nature Communications 9: 5185. 10.1038/s41467-018-07696-8 PMC6281677 30518761

[nph70176-bib-0020] Park HJ , Kim W‐Y , Park HC , Lee SY , Bohnert HJ , Yun D‐J . 2011. SUMO and SUMOylation in plants. Molecules and Cells 32: 305–316. 21912873 10.1007/s10059-011-0122-7 PMC3887640

[nph70176-bib-0021] Ross PL , Huang YN , Marchese JN , Williamson B , Parker K , Hattan S , Khainovski N , Pillai S , Dey S , Daniels S *et al*. 2004. Multiplexed protein quantitation in *Saccharomyces cerevisiae* using amine‐reactive isobaric tagging reagents. Molecular & Cellular Proteomics 3: 1154–1169. 15385600 10.1074/mcp.M400129-MCP200

[nph70176-bib-0022] Rytz TC , Feng J , Barros JAS , Vierstra RD . 2023. *Arabidopsis*‐expressing lysine‐null SUMO1 reveals a non‐essential role for secondary SUMO modifications in plants. Plant Direct 7: e506. 37465357 10.1002/pld3.506 PMC10350450

[nph70176-bib-0023] Rytz TC , Miller MJ , McLoughlin F , Augustine RC , Marshall RS , Juan Y , Charng Y , Scalf M , Smith LM , Vierstra RD . 2018. SUMOylome profiling reveals a diverse array of nuclear targets modified by the SUMO ligase SIZ1 during heat stress. Plant Cell 30: 1077–1099. 29588388 10.1105/tpc.17.00993 PMC6002191

[nph70176-bib-0024] Sang T , Xu Y , Qin G , Zhao S , Hsu C‐C , Wang P . 2024. Highly sensitive site‐specific SUMOylation proteomics in Arabidopsis. Nature Plants 10: 1330–1342. 39294263 10.1038/s41477-024-01783-z

[nph70176-bib-0025] Srivastava M , Sadanandom A , Srivastava AK . 2021. Towards understanding the multifaceted role of SUMOylation in plant growth and development. Physiologia Plantarum 171: 77–85. 32880960 10.1111/ppl.13204

[nph70176-bib-0026] Tammsalu T , Matic I , Jaffray EG , Ibrahim AFM , Tatham MH , Hay RT . 2014. Proteome‐wide identification of SUMO2 modification sites. Science Signaling 7: rs2. 24782567 10.1126/scisignal.2005146 PMC4051997

[nph70176-bib-0027] Tammsalu T , Matic I , Jaffray EG , Ibrahim AFM , Tatham MH , Hay RT . 2015. Proteome‐wide identification of SUMO modification sites by mass spectrometry. Nature Protocols 10: 1374–1388. 26292070 10.1038/nprot.2015.095

[nph70176-bib-0028] Thompson A , Schäfer J , Kuhn K , Kienle S , Schwarz J , Schmidt G , Neumann T , Hamon C . 2003. Tandem mass tags: a novel quantification strategy for comparative analysis of complex protein mixtures by MS/MS. Analytical Chemistry 75: 1895–1904. 12713048 10.1021/ac0262560

[nph70176-bib-0029] Udeshi ND , Mertins P , Svinkina T , Carr SA . 2013. Large‐scale identification of ubiquitination sites by mass spectrometry. Nature Protocols 8: 1950–1960. 24051958 10.1038/nprot.2013.120 PMC4725055

[nph70176-bib-0030] Verma V , Croley F , Sadanandom A . 2018. Fifty shades of SUMO: its role in immunity and at the fulcrum of the growth–defence balance. Molecular Plant Pathology 19: 1537–1544. 29024335 10.1111/mpp.12625 PMC6637990

[nph70176-bib-0031] Vu LD , Gevaert K , De Smet I . 2018. Protein language: post‐translational modifications talking to each other. Trends in Plant Science 23: 1068–1080. 30279071 10.1016/j.tplants.2018.09.004

[nph70176-bib-0032] Wik L , Nordberg N , Broberg J , Björkesten J , Assarsson E , Henriksson S , Grundberg I , Pettersson E , Westerberg C , Liljeroth E *et al*. 2021. Proximity extension assay in combination with next‐generation sequencing for high‐throughput proteome‐wide analysis. Molecular & Cellular Proteomics 20: 100168. 34715355 10.1016/j.mcpro.2021.100168 PMC8633680

[nph70176-bib-0033] Zuniga NR , Frost DC , Kuhn K , Shin M , Whitehouse RL , Wei T‐Y , He Y , Dawson SL , Pike I , Bomgarden RD *et al*. 2024. Achieving a 35‐plex tandem mass tag reagent set through deuterium incorporation. Journal of Proteome Research 23: 5153–5165. 39380184 10.1021/acs.jproteome.4c00668 PMC12706455

